# TRandAugment: temporal random augmentation strategy for surgical activity recognition from videos

**DOI:** 10.1007/s11548-023-02864-8

**Published:** 2023-03-22

**Authors:** Sanat Ramesh, Diego Dall’Alba, Cristians Gonzalez, Tong Yu, Pietro Mascagni, Didier Mutter, Jacques Marescaux, Paolo Fiorini, Nicolas Padoy

**Affiliations:** 1 Altair Robotics Lab, University of Verona, 37134 Verona, Italy; 2 ICube, University of Strasbourg, CNRS, 67000 Strasbourg, France; 3 University Hospital of Strasbourg, 67000 Strasbourg, France; 4 IRCAD, 67000 Strasbourg, France; 5 Institute of Image-Guided Surgery, IHU Strasbourg, 67000 Strasbourg, France; 6 Fondazione Policlinico Universitario Agostino Gemelli IRCCS, 00168 Rome, Italy

**Keywords:** Data augmentation, Temporal augmentation, Surgical activity recognition, Temporal convolutional networks, Gastric bypass procedures, Cataract procedures

## Abstract

**Purpose:**

Automatic recognition of surgical activities from intraoperative surgical videos is crucial for developing intelligent support systems for computer-assisted interventions. Current state-of-the-art recognition methods are based on deep learning where data augmentation has shown the potential to improve the generalization of these methods. This has spurred work on automated and simplified augmentation strategies for image classification and object detection on datasets of still images. Extending such augmentation methods to videos is not straightforward, as the temporal dimension needs to be considered. Furthermore, surgical videos pose additional challenges as they are composed of multiple, interconnected, and long-duration activities.

**Methods:**

This work proposes a new simplified augmentation method, called *TRandAugment*, specifically designed for long surgical videos, that treats each video as an assemble of temporal segments and applies consistent but random transformations to each segment. The proposed augmentation method is used to train an end-to-end spatiotemporal model consisting of a CNN (ResNet50) followed by a TCN.

**Results:**

The effectiveness of the proposed method is demonstrated on two surgical video datasets, namely Bypass40 and CATARACTS, and two tasks, surgical phase and step recognition. *TRandAugment* adds a performance boost of 1–6% over previous state-of-the-art methods, that uses manually designed augmentations.

**Conclusion:**

This work presents a simplified and automated augmentation method for long surgical videos. The proposed method has been validated on different datasets and tasks indicating the importance of devising temporal augmentation methods for long surgical videos.

## Introduction

In the context of computer-assisted interventions, reliable recognition of surgical activities is a fundamental component that could allow automatic analysis of the surgical workflow by providing the valuable semantic information required to support clinical decisions, generate reports, and annotate data [1, 2]. These support systems could reduce surgical errors, increase patient safety, and help establish effective and efficient communication protocols [1–3]. Following the classification proposed in [4, 5], surgical procedures can be divided into surgical activities at different levels of granularity: phases, steps, actions, and motions. Surgical phases are described as a set of surgical aims to be executed for successfully completing the surgical procedure, while steps are defined as a set of surgical actions that need to be carried out to complete a surgical phase. These different activities are annotated as temporal segments of the procedure. Moreover, when performing minimally invasive surgeries, a change in the viewpoint on the anatomy may be required for executing each individual activity.

Previous research studies have tackled the problem of surgical activity recognition by capitalizing on videos recorded during surgery [6–12]. Many of these works have proposed deep learning models to extract spatial and temporal information from videos. All these methods employ a convolutional neural network (CNN) for visual feature learning followed by hidden Markov models (HMMs) [6], recurrent neural networks (RNNs) [7], long short-term memory (LSTMs) [8], temporal convolutional networks (TCNs) [9, 10], or transformers [11, 12] for temporal feature learning. Although deep learning models have been successfully used for tackling activity recognition in surgeries, training these models requires large volumes of data and an arduous effort for selecting hyperparameters.

One of the most essential components to be considered while training these models is data augmentation. Data augmentation is a commonly used method to generate additional data for improving the training of data-intensive deep learning models for image classification [13–15], object detection [16, 17], instance segmentation [16, 18], etc. Additionally, augmentation has been shown to have an impact on model robustness [19] and performance on semi-supervised and self-supervised learning methods [20–23]. However, specific augmentation policies need to be designed to capture prior knowledge for each domain, which requires expertise and manual work, making data augmentation methods difficult to extend to other domains and applications [14, 15, 24]. To tackle the challenge of manually designing augmentation policies, the latest research papers have proposed reinforcement learning to learn optimal policies [14, 15]. Recently, a simplified and more practical method (called RandAugment [24]) was proposed for addressing new difficulties, e.g., defining a proxy task and training on it, searching over 30 parameters, that arise with these automated data augmentation methods. Although the advances in automated data augmentation methods have been significant, these methods have been specifically developed for still images. Recently, a few augmentation methods specifically designed for video have been proposed  [17, 25–27]. These methods have proposed inserting temporal perturbations successionally to the video frames [25] or objects (obtained through instance segmentation) from one video onto another [17]. A learning-based method has been proposed in [26] that finds a pair of similar videos and then places objects from one video onto another video’s background. In [27], augmentation is applied to video frames ensuring smooth changes in its magnitude based on Fourier sampling. However, automated augmentation methods for videos have been unexplored.

In training video-based surgical activity recognition methods, previous works have used manually selected augmentations: horizontal flip [8, 10], rotations [9, 10], random cropping [8], translation [9], scale [9], and color jitter [10, 12]. These specific augmentation policies have been applied at the image level to train backbone CNNs. On the other hand, no effort has been made to propose augmentation approaches for surgical videos. The temporal dimension in videos assumes particular importance in activity recognition as intraoperative surgical videos are of longer duration compared to videos examined in the computer vision community, and they capture the complete surgical procedure composed of multiple complex activities. This temporality present in both surgical videos and activities needs to be considered and exploited, while designing augmentation policies for training spatiotemporal models.

To this end, the paper introduces a new simplified and automated data augmentation method, called *TRandAugment*, that aims to incorporate the essential temporal dimension. Inspired by work [24], the *TRandAugment* method proposes a compact and simple parameterization consisting of only 3 parameters, where one parameter is dedicated to the temporal dimension. *TRandAugment* is extensively evaluated on the task of surgical activity recognition at two levels of granularity, i.e., phase and step [4], using two large surgical video datasets: Bypass40 [10] and CATARACTS [28].

## Methodology

**Fig. 1:**
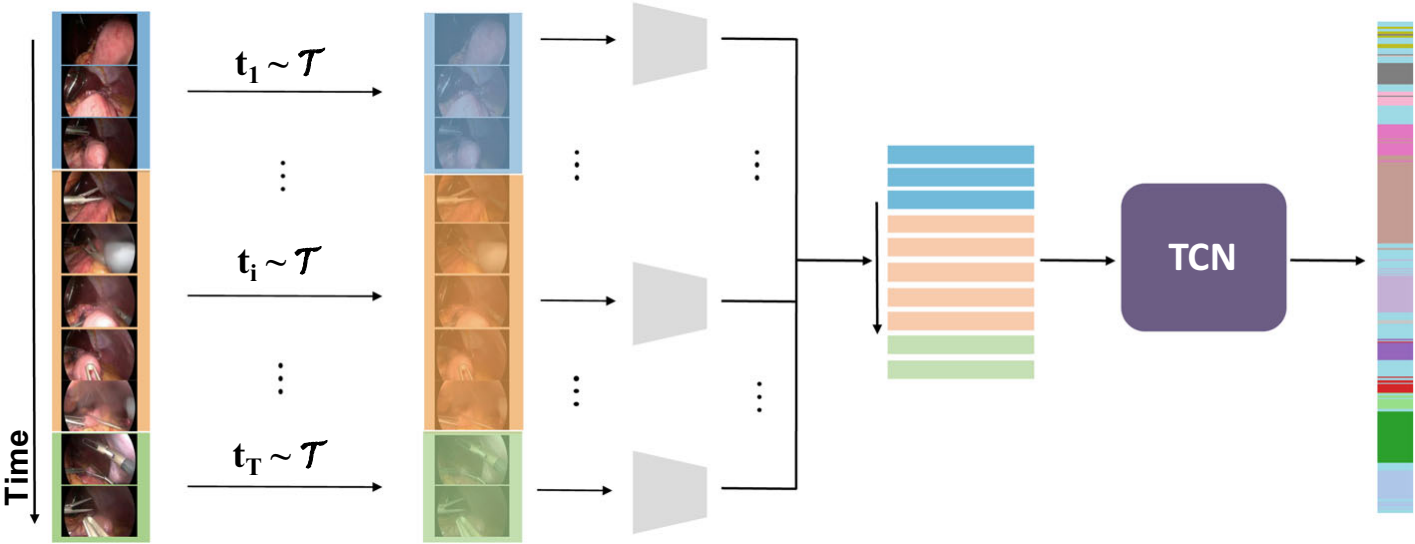
Pictographical representation of TRandAugment. A video is segmented into T clips, and a random augmentation $t_i$, sampled from a list of transforms $\tau$, is applied to clip i. The augmented clips are merged back to form a new video which is passed as input while training an end-to-end CNN+TCN network that predicts phases or steps

Automated activity recognition methods aim to segment endoscopic videos into surgical activities, i.e., phase or step. To improve the generalizability of activity recognition methods based on deep learning, this section introduces the proposed augmentation method, called TRandAugment, and the spatiotemporal model used to evaluate the method.

### TRandAugment

The goal of TRandAugment is to incorporate the temporal dimension present in surgical videos into the data augmentation methods for improving the generalization of activity recognition models. In pursuing this goal, we also want to propose a simplified and automated data augmentation method. Given that a recent method [24] operates only on a two-parameter space (*M*, *N*) compared to learned augmentation methods with over 30 parameters [14, 15], TRandAugment is designed to require only 3 parameters, where the first two adopt the same parameterization used in [24], while the third additional parameter *T* is used to characterize the temporal dimension. Similar to previous works [14, 24], a set $\tau$ of 10 transformations is utilized and applied with uniform probability $\frac{1}{\vert \tau \vert }$: 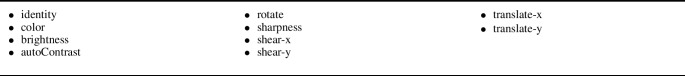


The choice of $\vert \tau \vert =10$ transformations is selected based on the domain knowledge of possible transformations that occur in endoscopic videos. Thus, we have excluded all the augmentations that, when applied, result in drastically different looking images that are highly unlikely to arise in surgical videos, such as posterize, solarize and equalize used in [24] and other novel augmentations proposed in the literature: YOCO [29], MixUp [30], CutMix [31] or AugMix [32].

As schematically represented in Fig. 1, the idea of TRandAugment is to apply different transformations to different temporal video segments. Thus, parameter *T* is introduced to control the number of temporal segments. Each video is split into a random $T'\in [1, T]$ segments, and for each segment *i* ($i\in [1, T']$), a random set of *N* transformation $\left\{ t_{i,1},...,t_{i, N} \vert \ t_{i,j}\sim \tau \right\}$ is applied uniformly on all the frames of that segment. The strength of each transformation is represented by magnitude *M* and linearly scaled between its minimum and maximum values mapped to an arbitrarily chosen integer scale from 0 to 30.

To maintain a notation consistent with previous methods, in particular [24], the proposed method is parameterized as (*M*, *N*, *T*), where *M* and *N* are defined as the magnitude and number of transformations to apply per segment, and *T* is the maximum number of temporal segments.

### Spatiotemporal model

The spatiotemporal model is comprised of ResNet-50 backbone, for visual feature learning, followed by a single-stage TCN (SS-TCN), for temporal modeling. The presented model is a powerful architecture comparable to other recent state-of-the-art methods [9–12]. Furthermore, it is modular and can easily accommodate new spatial and temporal models that could be proposed for activity recognition. This model is used in all the experiments and is trained end to end for the task of surgical activity recognition considering both phases and steps.

ResNet-50 [33] has been a popular model of choice in many recent works on phase/step recognition [8–10, 34]. The model is also employed in this work for visual feature learning. For long temporal modeling, TCNs have been shown to outperform RNNs [9, 10]. A single-stage model is employed over a multi-stage. This is motivated by the work of [10] where the multi-stage did not show improvements over the single-stage for both phase and step recognition. SS-TCN consists of only temporal convolutional layers that perform causal convolutions, which depend only on the current and *n* previous frames designed for online recognition.

The spatiotemporal model takes as input a video containing $\Upsilon$ frames $x_{1:\Upsilon }$. ResNet-50 extracts visual features of size $f=2048$ from $224\times 224\times 3$ RGB images. The frame-wise features are stacked over time for the TCN model, which outputs predictions $\hat{y}_{1:\Upsilon }$, where $\hat{y}_i$ is the class label for the current timestamp *i*, $i\in [1,\Upsilon ]$. Since both the tasks at hand (phase and step) are multi-class classification problems with imbalance in class distribution, following [9, 10], a class-weighted cross-entropy loss is used.

## Experimental setup

### Datasets

#### Bypass40 (BY40)

The *Bypass40* dataset [10], courteously shared by the authors of [10], comprises 40 Laparoscopic Roux-en-Y gastric bypass (LRYGB) procedures with average video duration of 1 h and 45 min. The complex workflow of LRYGB surgeries is represented with 11 phases and 44 steps, and the dataset is fully annotated with both these types of activities defined at different levels of granularity. A full list of all the phases and steps is presented in [10]. All the videos have a resolution of $854 \times 480$ or $1920 \times 1080$ pixels and are recorded at 25 fps. Following the same data split as [10], the dataset has been segregated into 24, 6, and 10 videos for training, validation, and test sets, respectively. The frames have been extracted at 1 fps and resized to ResNet-50’s input size of $224\times 224$.

#### CATARACTS (CA50)

The CATARACTS dataset^1^ [28, 35] consists of 50 videos of cataract surgical procedures. The dataset is annotated per frame with only steps as part of the CATARACTS2020 challenge. A complete list of all 19 steps is tabulated on the challenge website.^2^ The 50 videos are split into 25, 5, and 20 subsets for training, validation, and test sets, respectively. Frames are extracted at 1 fps and resized from $1920\times 1080$ to $224\times 224$.

### Training and evaluation

#### Baselines

TRandAugment, or TRA, is compared against different baselines. RandAugment [24], referred to as RA, is the first comparison where the augmentations are applied independently for each image in a video. Next, RandAugment is extended to UniformRandAugment, called URA, where augmentation is applied uniformly on all the frames in a video. TRA is a more generalized method encapsulating both RA and URA, where setting $T=1$ reduces TRA to URA, while $T=\Upsilon$ ($\Upsilon$: number of frames in a video) transforms TRA to RA. Finally, all the methods are compared against the state-of-the-art MTMS-TCN [10] that used a manually designed ‘Custom’ set of augmentations (flip, saturation, rotation) for surgical activity recognition.

**Table 1:** The use of temporally consistent augmentations does matter: RA vs URA. All results are reported on the validation set on the CA50 dataset for step recognition

M	$\vert \tau '\vert$	RA		URA	
		ACC	F1	ACC	F1
15	3	74.63	58.75	76.81	63.73
15	5	70.10	54.35	75.75	64.43
15	9	73.31	61.21	76.20	62.80
15	Avg	72.68	58.10	76.25	63.65
30	3	**77**.**31**	**64**.**62**	78.05	66.88
30	5	69.66	54.48	78.45	66.99
30	9	70.70	53.87	**79**.**74**	**68**.**07**
30	Avg	72.55	57.66	78.75	67.31

Bold values indicate the best performance

#### Training

In all the experiments, the ResNet-50 backbone model is initialized with ImageNet pretrained weights. Then, the complete ResNet-50 + SS-TCN model is trained in an end-to-end fashion for the task of phase/step recognition. To train the TCN, which requires temporal information, features from all the past frames in the video are cached by utilizing a feature buffer. This feature buffer is reset at the end of the video. The spatiotemporal model is trained for 50 epochs with a learning rate of 1e-5 and a batch size of 64. The proposed method and model have been implemented in PyTorch, and the experiments ($\sim$ 3500 GPU hours) were trained on NVIDIA RTX 6000 and V100 GPUs.

#### Evaluation

The effectiveness of the method is measured using accuracy (ACC), precision (PR), recall (RE), and F1-score (F1) metrics. The metrics are computed per video (averaged across classes) and are averaged across all the videos in the given set, following the same evaluation protocol as [9–11, 23].

## Results and discussion

In this section, we analyze the different components that influence the design of *TRandAugment*. Initially, we study the importance of temporally consistent augmentations in Sect. “Do temporally consistent augmentations matter?”, then we analyze the impact of parameter *M* in Sect. “Effect of magnitude (M)”, the number of transformations in Sect. “Do all augmentations help?” and impact of the parameter *T* in Sect. “Impact of parameter T on TRA”. Finally, we present the performance of the proposed method considering the optimal parameters on both datasets (Sect. “TRandAugment”).

### Do temporally consistent augmentations matter?

One of the key differences between videos and images is the additional temporal dimension. An obvious question is to study the importance of temporally consistent augmentations when training models on videos. To study the effect of temporal consistency, Table 1 compares the image-based augmentation method, RA, against the temporally consistent URA method on the CATARACTS dataset. The comparison is carried out at different settings ($M=\{15,30\}$, $N=1$, $\tau '\subset \tau :\vert \tau '\vert =\{3,5,9\}$). URA consistently performs better than RA in all the settings. Furthermore, the mean of RA, when averaged across $\vert \tau '\vert$ at both settings of $M=\{15,30\}$, is $\sim$3–7% below the best-performing model compared to URA ($\sim$1%). This indicates the instability of RA due to its policy of independent frame-wise augmentation, which breaks temporal visual consistency. Interestingly, the best RA model is obtained by utilizing a smaller set of augmentations $\vert \tau \vert =3$, which indicates that the model can learn significantly better when there is less variance in image appearance temporally. All the observations confirm that temporally consistent augmentations are important when training spatiotemporal models.

### Effect of magnitude (M)

To study the effect of augmentation magnitude, Table 2 compares model performance over various settings of $M=\{5,10,15,20,30\}$ for URA and TRA while keeping all other parameters fixed ($\vert \tau '\vert =5$, $N=1$, $T=5$). Both URA and TRA show higher performance at higher magnitudes with the best results obtained at $M=30$ on both tasks and datasets. Irrespective of the augmentation method used, higher magnitudes seem to have a direct effect on the performance of the model for different tasks and datasets. However, we notice that TRA performance is below URA at $M=30$. This is not a valid comparison as the other parameters $\vert \tau '\vert$, *N*, and *T* are fixed and sub-optimal. Hence, we perform these experiments to solely study the effect of magnitude on URA and TRA independently. The full comparison of TRA against other methods is discussed in Sect. “TRandAugment”.

**Table 2:** Effect of magnitude M. All results are reported on the F1-score metric

M	CA50 - step		BY40 - phase		BY40 - step	
	URA	TRA	URA	TRA	URA	TRA
5	64.23	60.59	85.06	85.02	54.55	53.78
10	63.75	63.40	82.72	84.59	54.39	54.62
15	64.43	63.67	84.83	85.64	56.64	56.38
20	61.61	62.22	84.54	82.70	57.39	56.06
30	**66**.**99**	**64**.**56**	**87**.**71**	**86**.**18**	**58**.**70**	**59**.**34**

Bold values indicate the best performance

### Do all augmentations help?

To study the importance of using all the augmentations, Table 3 lists different experiments in terms of F1-score on the validation set, with $N=1$ and $T=5$, where subsets of transforms ($\tau '\subset \tau :\vert \tau '\vert =\{3,5,9\}$) are randomly sampled from $\tau$. For the task of step recognition on both datasets, the best model performances are obtained when all transforms are utilized. On the other hand, the model performs best at an intermediate $\vert \tau '\vert =5$ for recognizing phases for both settings of $M=\{15,30\}$. However, at a higher magnitude ($M=30$), the model performs equally well at $\vert \tau '\vert =10$ compared to $\vert \tau '\vert =5$ for phase recognition. In short, TRA benefits by utilizing all the transforms $\tau$.

**Table 3:** Influence of the set of augmentations. All results report the F1-score metric

$\vert \tau '\vert$	M	TRA		
		CA50 - step	BY40 - phase	BY40 - step
3	15	65.92	83.21	56.36
5	15	63.67	**85**.**64**	56.38
9	15	**66**.**81**	82.99	**57**.**65**
3	30	62.93	83.27	59.85
5	30	64.56	**86**.**18**	59.34
9	30	**68**.**66**	**86**.**10**	**60**.**92**

Bold values indicate the best performance

### Impact of parameter T on TRA

The key component of the proposed TRA method is the parameter *T* that captures the variance in the appearance of the frames across a video. TRA is inspected with different settings of parameter $T=\{1,3,5,8\}$ at two different magnitudes $M=\{15,30\}$ while fixing $N=1$ and $\vert \tau '\vert =10$. The results in Table 4 show that at $T=5, M=30$ the model achieves the best performance on all the different tasks and across the two datasets. This indicates that augmenting at the clip level benefits the training of activity recognition models and the proposed TRA parameterization (*M*, *N*, *T*) allows us to easily find optimal parameters.

**Table 4:** Impact of the number of temporal segments T with different augmentations on TRA. All results are reported on the F1-score metric on the validation set

T	M	F1		
		CA50 - step	BY40 - phase	BY40 - step
3	15	66.11	85.53	56.94
5	15	66.81	84.98	55.69
8	15	67.10	85.49	55.66
3	30	65.21	**86**.**16**	59.05
5	30	**68**.**66**	**86**.**22**	**60**.**47**
8	30	66.74	85.92	59.13

Bold values indicate the best performance

**Table 5:** Comparison of different methods on BY40 and CA50 test sets. * denotes models trained in a multi-task setup requiring additional phase/step labels

Dataset	Method	$\vert \tau '\vert$	M, N, T	ACC	PR	RE	F1
Task							
	Custom [10]	–	–, –, –	81.79 ± 12.30	77.82 ± 13.61	82.25 ± 14.69	78.21 ± 14.90
CA50	RA [24]	3	30, 1, –	80.45 ± 10.33	76.48 ± 13.00	81.34 ± 13.56	76.87 ± 14.01
Step	URA (ours)	10	30, 1, –	83.24 ± 10.64	77.04 ± 14.20	82.33 ± 14.68	78.02 ± 14.98
	TRA (ours)	10	30, 1, 5	**83.64 ± 10.67**	**78.38 ± 14.11**	**84.06 ± 14.18**	**79.43 ± 15.09**
	Custom* [10]	–	–, –, –	90.26 ± 6.44	84.74 ± 7.71	81.75 ± 9.12	81.31 ± 9.07
BY40	URA (ours)	10	30, 3, –	**93.55 ± 3.24**	83.25 ± 7.80	86.07 ± 7.61	83.51 ± 7.93
Phase	TRA (ours)	10	30, 2, 5	93.17 ± 4.27	**86.42 ± 8.50**	**86.70 ± 6.72**	**85.20 ± 8.40**
	Custom* [10]	–	–, –, –	75.46 ± 9.34	55.58 ± 9.88	52.78 ± 9.22	50.35 ± 9.75
BY40	URA (ours)	10	30, 2, –	80.55 ± 6.61	61.32 ± 8.11	62.13 ± 7.74	58.52 ± 8.46
Step	TRA (ours)	10	30, 2, 5	**80.80 ± 7.90**	**63.66 ± 9.08**	**63.94 ± 8.31**	**60.06 ± 9.22**

Bold values indicate the best performance per dataset/task per metric

### TRandAugment

Table 5 compares different augmentations methods on the test set with optimal parameters. As noticed earlier, temporally consistent augmentations are beneficial, and hence, both URA and TRA, which enforce this consistency, outperform image-level augmentation method RA by 1–2% in F1 and $\sim$3% in accuracy for the task of step recognition on CATARACTS. Additionally, URA and TRA both show improvement over the state-of-the-art MTMS-TCN model, which utilized a ‘Custom’ set of augmentations by 1–5% across all the metrics for phase recognition on Bypass40. We can further notice a significant improvement of 5–11% across all the metrics for recognizing steps on Bypass40. This improvement could be attributed to the larger set of transforms $\vert \tau \vert =10$.

TRA, on the other hand, outperforms URA on both the phase and step recognition tasks and both datasets. TRA achieves a 1–3% improvement in phase and step recognition on Bypass40 and CATARACTS, respectively. Moreover, for step recognition on Bypass40, TRA achieves a +2% and +1.5% improvement in precision and F1-score over URA. The performance improvement of the proposed TRA method over URA could be attributed to the temporally consistent augmentations applied at the clip level. TRA enables the extension of video datasets with videos composed of different segments augmented differently, which when used in training improves the generalization of deep learning models. Besides, the parameterization of TRA is independent of the underlying recognition task or dataset, which enables the proposed method to be applicable to other surgical procedures and tasks.

### Limitations

The (*M*, *N*, *T*) parameterization of *TRandAugment* simplifies the process of selecting a good augmentation policy, for training, that induces both spatial and temporal variations in the input videos. Yet, it does not completely eliminate the search for optimal parameters, which adds computational expense. Further studies are required to better understand if or when datasets or tasks may require a separate search to achieve optimal performance. Another drawback of *TRandAugment* is that it works only in the input space. Few works in the literature have proposed adding variations in the model’s feature space to improve generalizability [36, 37]. Unlike input space augmentations, designing feature space augmentations is extremely challenging because the domain or the noise characteristics of the feature space is not well-studied. Nevertheless, this could be an interesting extension to our proposed method, especially for training the temporal component of spatiotemporal models.

## Conclusion

This paper introduced a new augmentation method called *TRandAugment* that simplifies data augmentation pipelines. Given a video, creates pseudo-videos with different clips augmented differently. The method is parameterized with magnitude (M), the number of augments (N), and the number of temporal augments (T). This parameterization provides a simple framework to search for optimal configuration and operates at a level with significantly reduced search space, in line with current research in data augmentation. The proposed method has been validated on two large surgical video datasets, considering both the phase and step recognition tasks, obtaining a boost in the performances thus demonstrating the impact of *TRandAugment*. New open questions arise on how this method may improve model robustness [19], federated learning or semi-/self-supervised learning [20–23, 34]. Furthermore, the proposed method could be applicable to other tasks, such as tool localization and tracking [38], action triplets [39], and video semantic segmentation [40]. Future work will study the value of TRandAugment in these different settings and tasks.

## Footnotes

1 https://ieee-dataport.org/open-access/cataracts.

2 https://www.synapse.org/#!Synapse:syn21680292/wiki/601563.
